# Investigation of optimal display size for viewing T1‐weighted MR images of the brain using a digital contrast‐detail phantom

**DOI:** 10.1120/jacmp.v17i1.5876

**Published:** 2016-01-08

**Authors:** Hideki Fujita, Nao Kuwahata, Hiroyuki Hattori, Hiroshi Kinoshita, Haruyuki Fukuda

**Affiliations:** ^1^ Department of Radiation Oncology Osaka Saiseikai Nakatsu Hospital 2‐10‐39 Shibata Kita‐ku Osaka 580‐0012 Japan

**Keywords:** contrast‐detail phantom, display size, observer performance, image quality figure

## Abstract

We clarified the relationship between the display size of MRI images and observer performance using a digital contrast‐detail (d‐CD) phantom. The d‐CD phantom was developed using Microsoft Visual Basic 2010 Express. It had a 512×512 matrix in size and a total of 100 holes, whose diameter increased stepwise from 4 to 40 pixels with a 4‐pixel interval in the vertical direction; the contrast varied stepwise in the horizontal direction. The digital driving level (DDL) of the background, the width of the DDL, and the contrast were adjustable. These parameters were determined on the basis of the actual T1‐weighted magnetic resonance (MR) images of the brain. In this study, the DDL, width, and contrast were set to 85, 20, and 1, respectively. The observer performance study was performed for three different display sizes (30 cm×30 cm as the enlarged size, 16 cm×16 cm as the original size, and 10 cm×10 cm as the reduced size) using a 2‐megapixel color liquid crystal display monitor, and it was analyzed using Friedman and Wilcoxon statistical tests. The observer performances for the original display (p<0.01) and the reduced display sizes (p<0.01) were superior to that observed for the enlarged size, whereas there was no significant difference between the original display and reduced display sizes (p=0.31). Evaluation with the digital phantom simulating MR imaging also revealed that the original and reduced display sizes were superior to the enlarged display size in observer performance. The d‐CD phantom enables a short‐term evaluation of observer performance and is useful in analyzing relationship between display size and observer performance.

PACS number: 87.57.‐s

## I. INTRODUCTION

The use of picture archiving and communication system (PACS) and soft‐copy interpretation in radiology has become very popular. The diagnostic interpretations are influenced by various factors (e.g., workstation software, display devices, resolution, and luminance).^1^, ^2^, ^3^ The display size is also an important factor.^1^, ^4^

Contrast‐detail (CD) phantoms are capable of facilitating the visual evaluation of X‐ray images.^5^, ^6^, ^7^, ^8^ However, commercially available CD phantoms are primarily used for the evaluation of X‐ray images and difficult to use for CT or MRI images. We previously reported on the development of a d‐CD phantom that facilitates evaluation of observer performance, and investigated the relationship between display size and observer performance.^(4)^ Recently, we developed a new digital CD (d‐CD) phantom in which the digital driving level (DDL) of the background, width of the DDL, and contrast were adjustable. We clarified the relationship between the display size of the MRI images and observer performance using the newly developed d‐CD phantom.

## II. MATERIALS AND METHODS

### A. Development of the digital CD phantom

**Figure 1:**
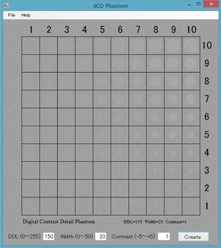
Appearance of the developed digital contrast‐detail phantom.

**Figure 2:**
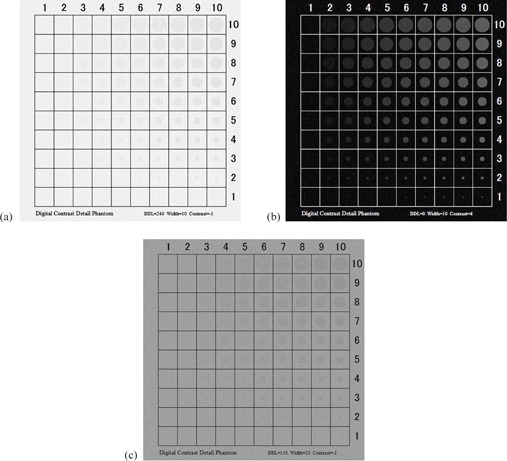
Sample of the produced digital contrast‐detail phantom: (a) DDL=240,Width=10,Contrast=−1; (b) DDL=0,Width=10,Contrast=1; (c) DDL=150,Width=20,Contrast=−1.

The d‐CD phantom was developed using Microsoft Visual Basic 2010 Express (Microsoft Corp., Redmond, WA) (Fig. 1). It had a 512×512 matrix and a total of 100 holes, whose diameter increased stepwise from 4 to 40 pixels with a 4‐pixel interval in the vertical direction; the contrast varied stepwise in the horizontal direction. “DDL” signifies the pixel value of the background in the d‐CD phantom. “Width” refers to the deviation of the background noise. “Contrast” signifies that the pixel value of the holes varies in a horizontal direction. When the “Create” button is pressed, a d‐CD phantom is produced. Figure 2 shows samples of the produced d‐CD phantom.

These parameters were determined on the basis of the actual T1‐weighted images (T1WIs) of the brain MRI. Ten patients for whom MRI examination was conducted at our medical institution were included in the present research. All brain MRI examinations were performed using a 1.5 T Signa HDxt scanner (GE Healthcare, Milwaukee, WI). The following scan parameters were employed for spin‐echo imaging: for T1WIs, TR/TE=600/11 ms, and bandwidth=97.7 Hz/pixel. In the imaging modes, the following were used: section thickness=5 mm,field of view=75 mm, two excitations, and acquisition matrix=288×224. In each of the 10 patients, signal intensities (SI) of normal cerebral white matter were measured on T1WIs without contrast agent administration. As the results of these analyzed data, the DDL, Width, and Contrast were set to 85, 20, and 1, respectively.

### B. Analysis of the observer performance using the d‐CD phantom

**Figure 3:**
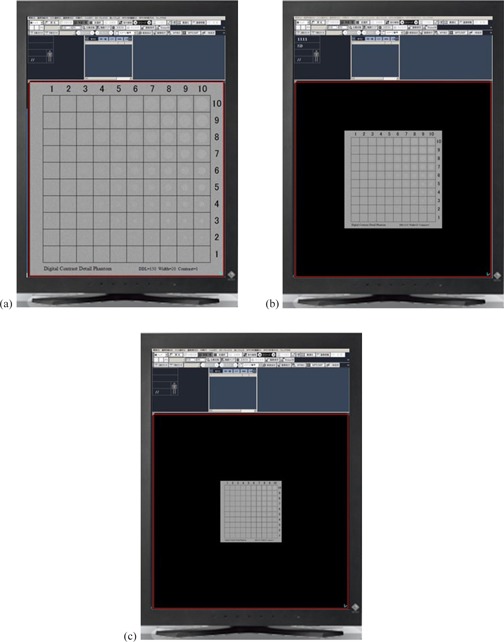
Extracted images on a 2‐megapixel color liquid crystal display monitor (DDL=85,Width=20,Contrast=1): (a) enlarged size: 30 cm×30 cm; (b) original size: 16 cm×16 cm; (c) reduced size: 10 cm×10 cm.

The observer performance study was performed for three different display sizes (30 cm×30 cm as the enlarged size, 16 cm×16 cm as the original size, and 10 cm×10 cm as the reduced size) (Fig. 3).

The observers read the extracted images on a 2‐megapixel color liquid crystal display (LCD) monitor (Radiforce RX240; EIZO, Ishikawa, Japan) using a DICOM Viewer (View R; Yokogawa, Tokyo, Japan). The actual maximum luminance of the LCD monitor was set at $400\;{\text{cd}/m}^{2}$, and the ambient lighting conditions were adjusted to 65 lux, while the display function of the monitor was set at a perceptually linearized curve (calibrated using the grayscale standard display function [GSDF]).^9^, ^10^ The GSDF has allowed radiologists to obtain a consistent image display from all digital modalities, except film digitizers.

For the image evaluation, the smallest visible holes were calculated from the average values for all the observers and plotted to form the CD curve. The quantification was performed by calculating an image quality figure (IQF).^7^, ^11^, ^12^ The IQF is defined as the sum of the products of contrast ($C_{i}$) and diameter ($D_{i}$) of the recorded visible objects, indicating a better image quality for lower values.

(1) $$IQF=\sum_{i=1}^{10}\left(Ci\times Di\right)$$

Ten radiation therapists observed the d‐CD phantom at a distance of 60 cm and determined the smallest visible holes. The results were analyzed using the Friedman and Wilcoxon statistical tests.

## III. RESULTS

**Figure 4:**
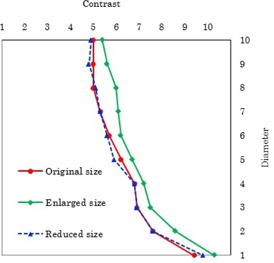
Contrast‐detail curve for visual evaluation of images in the three different viewing sizes.

The observer performances for the original display (p<0.01) and the reduced display sizes (p<0.01) were superior to that observed for the enlarged size, whereas there was no significant difference between the original display and reduced display sizes (p=0.31) (Table 1). Figure 4 shows the CD curve for visual evaluation.

**Table 1:** IQF values of all the observers for each display size.

	*Display Size*		
*Observer*	*Original*	*Enlarged*	*Reduced*
1	367	390	375
2	284	311	291
3	338	408	401
4	369	360	331
5	232	282	232
6	305	347	273
7	255	352	254
8	300	294	289
9	284	291	256
10	364	411	359
Mean (SD)	309.8 (48.3)	344.6 (48.6)	306.1 (57.3)

IQF=image quality figure; SD=standard deviation.

## IV. DISCUSSION

We developed a new d‐CD phantom that facilitates evaluation of observer performance. The simulation of phantoms corresponding to a variety of modalities is simple, as our d‐CD phantom can provide various signal conditions. We believe that use of our method employing a d‐CD phantom is advantageous because it allows image noise, sharpness, and contrast to be evaluated in a comprehensive manner, while accounting for the observer's perception.

In this study, we simulated the T1‐weighted MRI images of the brain using the d‐CD phantom and evaluated the observer performance for three different display sizes. Our investigation revealed that the CD curves of the original display and reduced display sizes are close to each other, and that those of the original display and enlarged display sizes are far from each other with no dependence on the diameter.

In a previous report,^(4)^ we evaluated the impact of display size on observer performance in high‐resolution CT images. We reported that the original display and reduced display sizes were superior in observer performance to the enlarged display size. Those findings agree with ours obtained in this study. When down‐sampling noisy images, the extended region considered in the interpolation also helps to reduce noise in the presentation.^(13)^ Therefore, we believe that there was no significant difference between the original display and the reduced display sizes. However, down‐sampling images with interpolation may not reveal subtle details seen at actual size because the human visual system has maximum contrast sensitivity at about 0.5 cycles/mm.^(13)^ Therefore, we believe that the original display size gives a better detection performance. In any case, display size should be recognized as an important factor in the soft‐copy diagnosis.

There are several limitations in our study. Although good visualization of the full scene is achieved when the diagonal display distance is about 80% of the viewing distance,^(13)^ the distance of observation was unrestricted in our study. In addition, we did not mention the relationship between the noise and the observer performance. In future, we intend to evaluate the influence of other factors on the observer performance. In our d‐CD phantom study, we simulated only the T1‐weighted MR images of the brain. This result may not be consistent with those of previous MR studies or different imaging modalities. However, it is easy to simulate phantoms corresponding to a variety of signal patterns by analyzing clinical data, because the DDL level, background noise, and contrast are adjustable.

Solid phantoms were introduced in several studies.^14^, ^15^ These phantoms have the following advantage, in that various types of images can be acquired for comparison by changing the image processing or scanning condition of the acquisition system. However, this advantage was a limitation for the digital phantom. Solid phantoms have the following disadvantage, in that their development takes a long time, and they are expensive owing to the material and labor costs.

On the other hand, the advantages of our proposed d‐CD phantom are as follows: phantom preparation is less expensive owing to lower material costs, and time is saved. The time required for observation was only 2– 3 min, and quantitative evaluation using IQF is possible.

In a previous study,^(16)^ we reported the usefulness of reduced display size in soft‐copy diagnosis by using clinical chest radiographs. However, the use of clinical data is time‐consuming. Therefore, by using a digital phantom, we were easily able to evaluate the relationship between display sizes and observer performance. The d‐CD phantom will save time and effort required for subjective evaluation during soft‐copy diagnosis.

## V. CONCLUSIONS

The evaluation with the digital phantom simulating MR imaging revealed that the original and reduced display sizes were superior to the enlarged display size in the observer performance. Based on these results, the original display size is recommended to optimize the soft‐copy reading for viewing MRI images.

The newly developed d‐CD phantom enables a short‐term evaluation of the observer performance, and is useful in analyzing the relationship between display size and observer performance.
